# The peptidoglycan-associated lipoprotein gene mutant elicits robust immunological defense in mice against *Salmonella enteritidis*

**DOI:** 10.3389/fmicb.2024.1422202

**Published:** 2024-06-05

**Authors:** Guixin Zhao, Wenlong Duan, Lu Zhang, Wenchao Sun, Wan Liu, Xiaoyu Zhang, Yanying Zhang, Qiumei Shi, Tonglei Wu

**Affiliations:** ^1^College of Animal Science and Technology, Hebei Normal University of Science and Technology, Qinhuangdao, China; ^2^Wenzhou Key Laboratory for Virology and Immunology, Institute of Virology, Wenzhou University, Wenzhou, China

**Keywords:** *Salmonella enteritidis*, Tol-Pal system, gene deletion, virulence, immune protection

## Abstract

**Background:**

*Salmonella enteritidis* (*S. enteritidis*), a zoonotic pathogen with a broad host range, presents a substantial threat to global public health safety. Vaccination stands as an effective strategy for the prevention and control of *S. enteritidis* infection, highlighting an immediate clinical need for the creation of safe and efficient attenuated live vaccines.

**Methods:**

In this study, a *S. enteritidis* peptidoglycan-associated lipoprotein (*pal*) gene deletion strain (Δ*pal*), was constructed. To assess its virulence, we conducted experiments on biofilm formation capability, motility, as well as cell and mouse infection. Subsequently, we evaluated the immune-protective effect of Δ*pal*.

**Results:**

It was discovered that deletion of the *pal* gene reduced the biofilm formation capability and motility of *S. enteritidis*. Cell infection experiments revealed that the Δ*pal* strain exhibited significantly decreased abilities in invasion, adhesion, and intracellular survival, with downregulation of virulence gene expression, including *mgtC*, *invH*, *spvB*, *sipA*, *sipB*, *ssaV*, *csgA*, and *pipB*. Mouse infection experiments showed that the LD_50_ of Δ*pal* increased by 10^4^ times, and its colonization ability in mouse tissue organs was significantly reduced. The results indicated that the *pal* gene severely affected the virulence of *S. enteritidis*. Further, immunogenicity evaluation of Δ*pal* showed a significant enhancement in the lymphocyte transformation proliferation capability of immunized mice, producing high titers of specific IgG and IgA, suggesting that Δ*pal* possesses good immunogenicity. Challenge protection tests demonstrated that the strain could provide 100% immune protection against wild-type strains in mice.

**Discussion:**

This study proves that the *pal* gene influences the virulence of *S. enteritidis*, and Δ*pal* could serve as a candidate strain for attenuated live vaccines, laying the foundation for the development of attenuated live vaccines against *Salmonella*.

## Background

*Salmonella* is a facultative intracellular pathogen that is Gram-negative and capable of infecting a variety of animals, including humans, severely endangering the health development of the global aquaculture industry and public health safety, and causing substantial economic losses (Ferrari et al., 2019). This bacterium has numerous serotypes, with over 2,600 serotypes reported to date (Jajere, 2019), with serotypes mainly infecting humans being *Salmonella enteritidis* (*S. enteritidis*) and *Salmonella typhimurium*, accounting for 40% of human cases of salmonellosis (Cao et al., 2023). Humans may contract *S. enteritidis* through the consumption of contaminated pork, beef, poultry, and eggs, or through exposure to fecal matter in areas lacking adequate sanitation. The manifestation of salmonellosis in humans includes symptoms such as abdominal pain, diarrhea, nausea, vomiting, fever, and headaches.

Antibiotics represent a widespread approach for bacterial infection treatment, yet they also play a role in environmental pollution and the emergence of multidrug-resistant strains, posing additional risks to human health. Vaccination is recognized as another crucial measure for preventing and managing *Salmonella* infections (Ruvalcaba-Gómez et al., 2022). *Salmonella* vaccines mainly include attenuated live vaccines, subunit vaccines, inactivated vaccines, and DNA vaccines. Due to the facultative intracellular nature of *Salmonella*, strong cellular immunity plays a crucial role in pathogen clearance, making attenuated live vaccines considered more effective in immune protection than other types of vaccines (Li et al., 2021; Jiang et al., 2022). Attenuated live vaccines can induce systemic immune responses, activating immune cells such as B cells and T cells, and promoting them to secrete cytokines like IL-1β, IL-2, IL-4, IL-6, IL-10, TNF-α, and IFN-γ. IL-1β plays a critical role in the regulation of immune and inflammatory responses to infections or sterile insults. IL-2 stimulates the immune response by promoting the development of T regulatory cells and enhancing the cytotoxic activity of natural killer cells. IL-4 is a cytokine that is key in regulating immune responses, particularly in promoting the differentiation of naive helper T cells (Th0 cells) into Th2 cells. IL-6 is a multifunctional cytokine that plays roles in inflammation, immune response, and hematopoiesis. IL-10 is an anti-inflammatory cytokine that functions to limit immune responses to pathogens and prevent damage to the host by inhibiting the production of pro-inflammatory cytokines. TNF-α is a pro-inflammatory cytokine produced mainly by macrophages. TNF-α plays a role in apoptosis, inflammation, and the immune response to cancer and infection. IFN-γ is critical for innate and adaptive immunity against viral and intracellular bacterial infections and for tumor control (Kak et al., 2018; Ullrich et al., 2021; Behzadi et al., 2022; Kung et al., 2022; Song et al., 2022; Albuquerque Pereira et al., 2023; Mukherjee et al., 2023). Moreover, studies show that attenuated live vaccines of *Salmonella* are low in virulence to the host and can induce a strong and lasting mucosal and humoral immune response (Tennant and Levine, 2015), effectively reducing bacterial adhesion and colonization. With the development of genetic engineering technologies, deleting virulence genes has become an important strategy in the development of attenuated live vaccines. Currently, various *Salmonella* gene knockout strains are used as live vaccines, for example, immunization with a *Salmonella* strain with the protein tyrosine phosphatase (*sptP*) gene deleted stimulated good immune protection in mice (Lin et al., 2017); deletion of the *rfbB* and *rffG* genes related to LPS synthesis in *Salmonella typhi* significantly reduced the colonization ability in mice and induced elevated levels of IgA and IgG specific for ST-OMPs, and high levels of cellular immune responses (TH1 immune response) and IFN-γ cytokines; using homologous serotype strains *Salmonella typhi* and heterologous serotype strains *Salmonella choleraesuis* for challenge, the immune protection rates in mice reached 100 and 40%, respectively (Zhou et al., 2023). In addition, attenuated vaccines prepared by deleting virulence genes *cpxR*, *lon*, and SPI2 were also effective in reducing the colonization of wild-type strains in chickens, providing good immune protection (Yin et al., 2015; Kamble and Lee, 2016).

Pal (Peptidoglycan-associated lipoprotein, Pal) is part of the Tol-Pal system, which is a multiprotein complex spanning the inner and outer membranes of Gram-negative bacteria. Changes in the Tol-Pal system typically disrupt bacterial cell membrane integrity, disturb outer membrane permeability, and make bacteria more sensitive to antimicrobial peptides and detergents (Godlewska et al., 2009). Studies on *Burkholderia mallei* (Dyke et al., 2020), *Brucella* (Chen et al., 2022), *Haemophilus ducreyi* (Fortney et al., 2000), and *Klebsiella pneumoniae* (Hsieh et al., 2013) show that deletion of the *pal* gene disrupts cell membrane integrity, reducing bacterial colonization ability in the host and affecting virulence. The *pal* gene is a conserved gene found in various bacteria, including *Salmonella*, but the function of the *pal* gene in *S. enteritidis* has not been clearly defined. Therefore, this study constructed a *S. enteritidis pal* gene deletion strain and analyzed the impact of the *pal* gene on the virulence of *S. enteritidis* through biofilm formation, antibiotic resistance, motility, *in vitro* stress, cell infection, and mouse infection experiments. Further, by conducting immunization and challenge protection experiments using KM mice, the immune protection effect of the *pal* gene deletion strain was analyzed. This study lays the foundation for the development of attenuated live vaccines for *S. enteritidis*.

## Materials and methods

**Figure 1:**
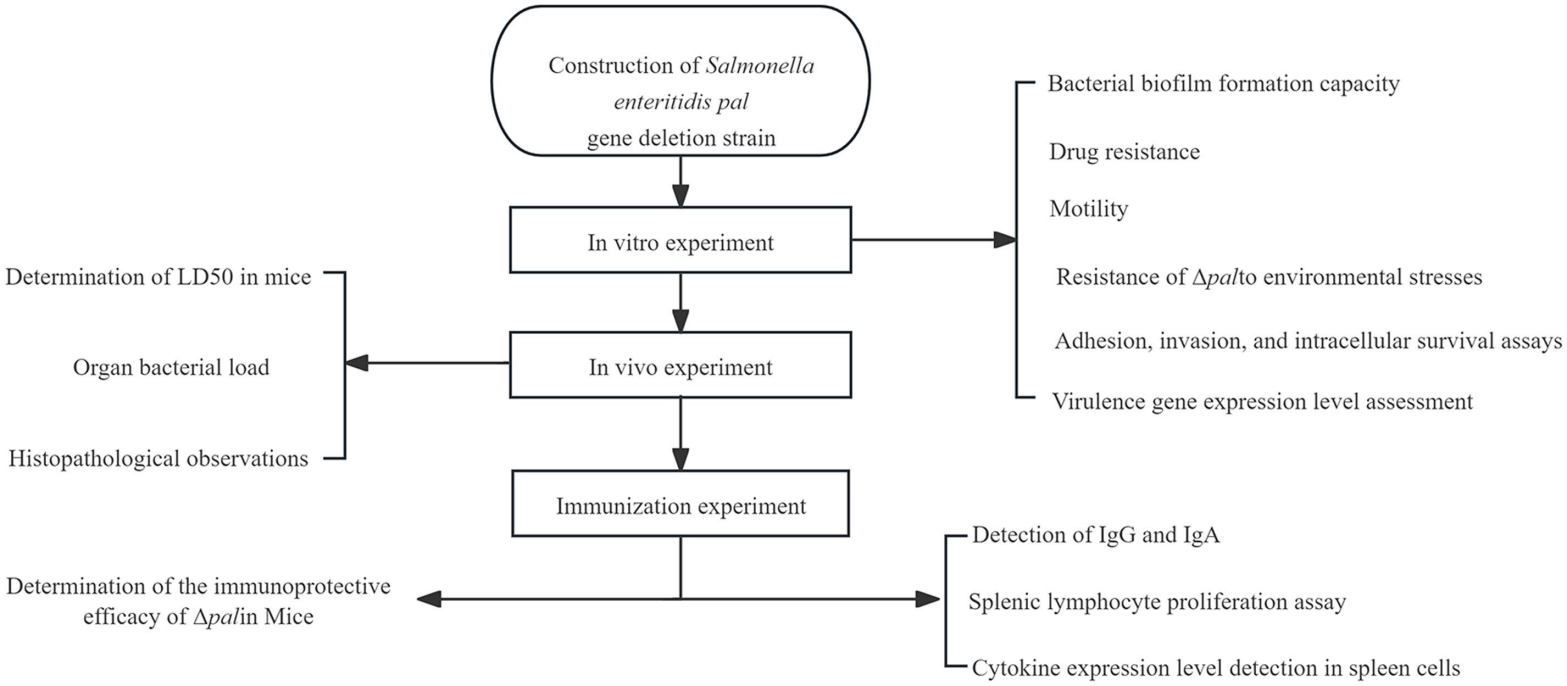
The flow chart of methodology section.

All experiments conducted in this study are illustrated in the flowchart (Figure 1).

### Experimental animal

Female Kunming (KM) mice, aged 6 to 8 weeks, were maintained in a sterile environment with standard feeding conditions throughout the experimental duration. The ambient temperature was consistently held at 22.0°C with a tolerance of ±0.5°C, and the relative humidity was maintained at 60% with a permissible variation of ±10%. A 12-h light/dark cycle was established for the housing conditions. Mice were obtained from Spay (Beijing) Biotechnology Co., Ltd., situated in Beijing, China. Ethical approval for the procurement and handling of the mice was obtained in strict compliance with the guidelines outlined in the Experimental Animal Regulation Ordinances by the Hebei Provincial Department of Science and Technology. This study was approved by the Animal Ethics Committee of Hebei Normal University of Science and Technology under permit number 2020-17.

### Bacterial strains

The bacterial strains and plasmids employed in this investigation are delineated in Table 1. Bacteria were cultured using LB liquid medium or plated on agar, with antibiotics added in accordance with the resistance characteristics of the strains, such as 100 μg/mL ampicillin and 34 μg/mL chloramphenicol. The human colorectal carcinoma epithelial Caco-2 BBE cell line and the murine macrophage RAW264.7 cell line used in this study were sourced from the BeNa Culture Collection (Shanghai, China). RAW264.7 cells were cultivated in DMEM supplemented with 10% fetal bovine serum (FBS) (Thermo Fisher Scientific Co., Ltd., China), and Caco-2 BBE cells were cultured in DMEM enriched with 20% FBS (Thermo Fisher Scientific Co., Ltd., China). Antibiotics were added when necessary, including 50 μg/mL streptomycin and 50 U/mL penicillin, or 100 μg/mL gentamicin, or 10 μg/mL gentamicin. All cells were incubated at 37°C in a 5% CO_2_ atmosphere.

**Table 1:** Wild-type and mutant strains and plasmids used for this study.

Strains or plasmids	Description	Source and/or reference
*S. enteritidis*		
C50336	Wild-type	Chinese National Institute for the Control of Pharmaceutical and Biological Products
Δ*pal*::Cat	First recombination strain	This study
Δ*pal*	*pal* deletion strain of C50336	This study
Δ*pal* + *pal*	The *pal* complementation strain of C50336	This study
Plasmids		
pKD3	FRT-Cm-FRT cassette; Cm^r^	This study
pKD46	containing the Red recombinase of λ-phage; Amp^r^	This study
pCP20	FLP recombinase, temperature sensitive replication; Amp^r^	This study
pMD-19 T	T-plasmid, Amp^r^	Takara
pMD-19 T -*pal*	The *pal* gene cloned into pMD-19 T	This study

### Construction of *S. enteritidis* pal gene deletion and complemented strains

The construction of the *S. enteritidis pal* gene deletion strain was carried out using the λ-Red homologous recombination technique as described in the literature (Datsenko and Wanner, 2000), with the procedure summarized as follows:

Primers P1 and P2 (Table 2) were used to PCR amplify a chloramphenicol resistance cassette flanked by *pal* gene homologous arms, using plasmid pKD3 as the template. The chloramphenicol cassette was electroporated into C50336 (containing plasmid pKD46) and cultured overnight at 37°C on LB agar plates containing chloramphenicol (34 μg/mL). The following day, single colonies were picked and shaken in LB broth containing chloramphenicol to grow, followed by PCR verification using primers P5 and P6. Positive colonies were cultured in LB broth, subjected to heat shock at 42°C to eliminate plasmid pKD46, and named Δ*pal*::Cat. Plasmid pCP20 was electroporated into the competent Δ*pal*::Cat cells to remove the chloramphenicol resistance gene, followed by overnight culture at 37°C on LB plates containing ampicillin (100 μg/mL). The next day, single colonies were picked and cultured in LB broth containing ampicillin, followed by PCR verification using primers P5 and P6, and heat shock at 42°C to eliminate plasmid pCP20, resulting in the strain named Δ*pal*.

**Table 2:** Primers used for the construction of the *pal* deletion mutant and complemented strain.

Primers	Primers sequences (5′ → 3′)
P1	CTAAAGGAATTAAAGAAATGCAACTGAACAAAGTGCTGAAGGGCCTGATGATTGCCCTGTGTGTAGGCTGGAGCTGCTTCG
P2	GTTACTGCTCATGCAATTCTCTTAGTAAACCAGTACAGCGCGACGGTTCTTAGCGTAAGCATATGAATATCCTCCTTAG
P3	TCAGATTCGTCAGATAACGGACGGC
P4	TTAGTAAACCAGTACAGCGCGACGG
P5	CCTGGTCGCCGTATCTGT
P6	TGAGTGACGCGGTCTTCG
P7	CAACAAGAACGCCAGCAA
P8	GCGAAGTCAGAACGGATA

To construct the complemented strain, primers P3 and P4 were used to amplify the *pal* gene complementation fragment from the genomic DNA of C50336, which was then cloned into pMD-19 T. The recombinant plasmid pMD-19 T-*pal* was electroporated into the competent Δ*pal* cells, with positive clones verified using primers P3 and P4 (Table 2) and named Δ*pal* + *pal*. RNA from C50336 and Δ*pal* was extracted using a bacterial RNA extraction kit (Aidlab, China), reverse transcribed into cDNA, and subjected to qPCR verification using primers P7 and P8 (Table 2) to assess the expression of the *pal* gene in C50336, Δ*pal*, and Δ*pal* + *pal*.

### Biofilm formation assay

Biofilm formation capabilities of C50336, Δ*pal*, and Δ*pal* + *pal* strains were assessed as follows: bacterial cultures of C50336, Δ*pal*, and Δ*pal* + *pal* were diluted 100-fold and 5 mL of each diluted culture was added to tubes, which were then incubated at 28°C for 72 h without disturbance. The tubes were washed with PBS, fixed with 6 mL of methanol for 15 min, washed again with PBS, stained with 2% crystal violet for 30 min, followed by a final PBS wash. The tubes were dried until no water spots were visible, and results were observed and documented through photography. In a 96-well microtiter plate, 200 μL/well of the diluted bacterial cultures of C50336, Δ*pal*, and Δ*pal* + *pal* were added and incubated at 28°C for 72 h without disturbance. The wells were washed with PBS, fixed with 250 μL of methanol per well for 30 min, washed again with PBS, stained with 250 μL of 2% crystal violet for 10 min, followed by a final PBS wash. Crystal violet was dissolved in 250 μL of anhydrous ethanol per well, and the OD_570_ was measured. Each experimental group was repeated three times.

RNA from strain C50336 and Δ*pal* was extracted using a bacterial RNA extraction kit (Aidlab, China), with DNA removal followed by reverse transcription into cDNA. Primers can be found in the references (Pande et al., 2016; Gupta et al., 2020; Zhang et al., 2024). The expression levels of biofilm-associated genes *fimD*, *csgD*, *bcsA*, *ompR*, and *rpoS* in Δ*pal* were detected using dye-based quantitative PCR (qPCR), Primer sequences and gene functions can be found in Table 3. The 16S rRNA gene served as an internal reference gene, and relative expression levels were calculated using the 2^−ΔΔCt^ method.

**Table 3:** Primers used for the qPCR detection of biofilm genes and virulence factors.

Genes	Function	Primers	Primers sequences (5′ → 3′)
*fimD*	Outer membrane usher protein FimD	Forward	CGCGGCGAAAGTTATTTCAA
		Reverse	CCACGGACGCGGTATCC
*csgD*	Biofilm	Forward	GCCTCATATTAACGGCGTG
		Reverse	AGCGGTAATTTCCTGAGTGC
*bcsA*	Osmolarity response regulator	Forward	GCCCAGCTTCAGAATATCCA
		Reverse	TGGAAGGGCAGAAAGTGAAT
*ompR*	Bacterial cellulose synthase	Forward	TGTGCCGGATCTTCTTCCA
		Reverse	CTCCATCGACGTCCAGATCTC
*rpoS*	Oxidative stress, RNA polymerase sigma factor RpoS	Forward	TTTTTCATCGGCCAGGATGT
		Reverse	CGCTGGGCGGTGATTC
*rfbH*	Lipopolysaccharide biosynthesis	Forward	ACGGTCGGTATTTGTCAACTCA
		Reverse	TCGCCAACCGTATTTTGCTAA
*mgtc*	Mg (2+) transport ATPase protein C	Forward	CGAACCTCGCTTTCATCTTCTT
		Reverse	CCGCCGAGGGAGAAAAAC
*invH*	Cell adherence/invasion protein	Forward	CCCTTCCTCCGTGAGCAAA
		Reverse	TGGCCAGTTGCTCTTTCTGA
*spvB*	Actin ADP ribosyltransferase 2C toxin SpvB	Forward	TGGGTGGGCAACAGCAA
		Reverse	GCAGGATGCCGTTACTGTCA
*sipA*	Pathogenicity island 1 effector protein	Forward	CAGGGAACGGTGTGGAGGTA
		Reverse	AGACGTTTTTGGGTGTGATACGT
*sipB*	Pathogenicity island 1 effector protein	Forward	GCCACTGCTGAATCTGATCCA
		Reverse	CGAGGCGCTTGCTGATTT
*ssav*	Secretion system apparatus protein SsaV	Forward	GCGCGATACGGACATATTCTG
		Reverse	TGGGCGCCACGTGAA
*csgA*	Pathogenicity island protein	Forward	AATGCCACCATCGACCAGTG
		Reverse	CAAAACCAACCTGACGCACC
*pipB*	Bacterial Adhesion and Invasive Capacity	Forward	GCTCCTGTTAATGATTTCGCTAAAG
		Reverse	GCTCAGACTTAACTGACACCAAACTAA
16S rRNA	Internal reference gene	Forward	CCAGGGCTACACACGTGCTA
		Reverse	TCTCGCGAGGTCGCTTCT

### Minimum inhibitory concentration assay for Δ*pal* with polymyxin B

In the first well of a 96-well microtiter plate, 200 μL of LB liquid medium containing 1 mg/mL polymyxin B was added. Wells from 2 to 12 were filled with 100 μL of LB liquid medium without polymyxin B. A serial two-fold dilution was performed by transferring 100 μL from the first well into the second, mixing thoroughly, and repeating the process through to the last well. To each well, 10 μL of a 10^9^ CFU/mL bacterial culture of C50336 or Δ*pal* was added, and the plate was incubated at 37°C for 48 h. Each experimental set was repeated three times. A turbid culture in the wells indicated bacterial growth, while the concentration of polymyxin B in the clear wells was considered the minimum inhibitory concentration (MIC).

### Motility assay

Referring to previous methods (Nikhil et al., 2022), Five microliters of C50336, Δ*pal*, and Δ*pal* + *pal* bacterial cultures were each stabbed vertically into LB agar medium containing 0.3% agar and incubated at 37°C for 6 h. The motility diameters of C50336, Δ*pal*, and Δ*pal* + *pal* were measured.

### Resistance of Δ*pal* to environmental stresses

According to the method described in the reference (Zhang et al., 2024), one milliliter of logarithmic phase bacterial culture of C50336 and Δ*pal* was centrifuged, washed, and resuspended in an equal volume for tenfold serial dilutions, followed by colony counting using the spread plate method (recorded as A). For acid and alkaline stress, C50336 and Δ*pal* cultures were added to physiological saline at pH 3.5 and pH 10.0, respectively, and incubated at 37°C for 1 h. For oxidative stress, the cultures were added to physiological saline containing H_2_O_2_ (10 mmol/L) and incubated at 37°C for 10 min. For thermal stress, the cultures were added to 0.85% physiological saline and incubated at 42°C for 1 h. Bacterial counts were conducted again (recorded as B). The bacterial survival rate was calculated as (B/A) × 100%.

### Adhesion, invasion, and intracellular survival assays

According to the previous methods (Zhang et al., 2020), Human colorectal carcinoma epithelial Caco-2 cells were seeded into two 12-well plates at a density of 3 × 10^5^ cells/well and incubated for 12 h. After washing with PBS, the cells were infected with bacterial cultures of C50336, Δ*pal*, and Δ*pal* + *pal* at a multiplicity of infection (MOI) of 100:1. Centrifugation at 1000 rpm for 10 min facilitated bacterial adhesion for the adhesion and invasion experiments. To determine adhesion capability, cells infected with bacteria were incubated for 1 h, washed with PBS, and treated with 0.5% Triton X-100 for 8 min. To assess invasion capability, after incubating the bacteria-infected cells for 1 h and washing with PBS, 1 mL of DMEM containing 20% FBS and gentamicin (100 μg/mL) was added per well and incubated for another hour, followed by washing with PBS and treatment with 0.5% Triton X-100 for 8 min. The lysates were diluted tenfold in PBS and plated on *Salmonella-Shigella* (SS) agar for colony counting. The adhesion and invasion rates of C50336, Δ*pal*, and Δ*pal* + *pal* were calculated using the formulae: Adhesion rate = (Number of adhered bacteria/Number of bacteria inoculated per well) × 100%; Invasion rate = (Number of intracellular bacteria/Number of bacteria inoculated per well) × 100%.

Referring to previous methods (Jung et al., 2022), RAW264.7 was seeded into two 12-well plates at a density of 3 × 10^5^ cells/well and incubated for 12 h. After washing with PBS, the cells were infected with bacterial cultures of C50336, Δ*pal*, and Δ*pal* + *pal* at an MOI of 100:1, followed by centrifugation at 1000 rpm for 10 min. After a 2-h incubation, the cells were washed with PBS to remove unadhered bacteria. Then, 1 mL of DMEM containing 10% FBS and gentamicin (100 μg/mL) was added and incubated for 1 h. After washing with PBS, 1 mL of DMEM containing 10% FBS and gentamicin (10 μg/mL) was added for further incubation for 1 and 20 h. Following another PBS wash, cells were treated with 0.5% Triton X-100 for 8 min. The cell lysates were diluted tenfold and plated on SS agar for colony counting. The intracellular survival rate of C50336, Δ*pal*, and Δ*pal* + *pal* within RAW264.7 cells was calculated using the formula: Intracellular survival rate = (Number of bacteria inside cells at 23 h/Number of bacteria inside cells at 3 h) × 100%.

### Virulence gene expression level assessment

RNA from strain C50336 and Δ*pal* was extracted using a bacterial RNA extraction kit (Aidlab, China), according to the manufacturer’s instructions, with subsequent DNA removal and reverse transcription into cDNA. Primers can be found in the references (Upadhyaya et al., 2013; Gupta et al., 2020; Zhang et al., 2024). The expression levels of virulence genes including *mgtC*, *invH*, *spvB*, *sipA*, *sipB*, *ssaV*, *csgA*, and *pipB* in strain C50336 and Δ*pal* were quantified using quantitative PCR (qPCR). Primers and gene functions can be found in Table 3. The 16S rRNA served as the internal reference gene, and relative expression levels were calculated using the 2^−ΔΔCt^ method.

### Determination of LD50 in mice

Referring to previous methods (Park et al., 2020), seventy-five 6 to 8-week female KM mice were divided into three groups of 25 mice each. The mice were intraperitoneally inoculated with C50336, Δ*pal*, or PBS. The inoculation doses for C50336 were 10^3^ CFU/mouse, 10^4^ CFU/mouse, 10^5^ CFU/mouse, 10^6^ CFU/mouse, and 10^7^ CFU/mouse. The inoculation doses for Δ*pal* were 10^6^ CFU/mouse, 10^7^ CFU/mouse, 10^8^ CFU/mouse, 10^9^ CFU/mouse, and 10^10^ CFU/mouse. Each dosage group contained five mice. The clinical symptoms of the mice were observed daily, and the number of deaths was recorded over a continuous 14-day period. The control group mice were intraperitoneally injected with an equal volume of PBS. The LD_50_ was calculated using a modified Karber’s method, with the formula as follows:

LD50 = 10^{Xk - i[p – (3-Pm-Pn) /4]}^, where i is the dose interval (i.e., the difference between the logarithmic doses of two adjacent dose groups); X_k_ is the logarithm of the maximum dose; p is the sum of the mortality rates for each dose group; Pm is the highest mortality rate; Pn is the lowest mortality rate.

### Bacterial burden determination in mouse tissue organs

Forty-five female KM mice were divided into three groups, with 15 mice in each group. Each group was intraperitoneally inoculated with the same dose of C50336, Δ*pal*, or PBS, at a dose of 2 × 10^6^ CFU/mouse. At 24, 72, and 120 h post infection, 5 mice infected with C50336 and Δ*pal*, respectively, were selected. The mice were euthanized under anesthesia, and their cecum, spleen, and liver were aseptically harvested. The cecum was washed three times with PBS and weighed. Homogenization in PBS was performed before bacterial counting on *Salmonella-Shigella* (SS) agar to calculate the bacterial load per gram of tissue (Log_10_CFU/g) for organ burden analysis. A control group was inoculated intraperitoneally with PBS.

### Analysis of mouse tissue pathological changes

Eighteen female KM mice, aged 6 to 8 weeks, were inoculated intraperitoneally with strain C50336 or Δ*pal*, six mice per group, at a dosage of 2 × 10^6^ CFU/mouse. At 48 and 72 h post infection, 3 mice infected with C50336 and Δ*pal*, respectively, were selected. The mice were euthanized under anesthesia, and their spleen, liver, and jejunum were aseptically collected. The contents of the jejunum were washed away with PBS. The spleen, liver, and jejunum were fixed in 4% paraformaldehyde (Biosharp, China), embedded in paraffin, sectioned, and stained with hematoxylin and eosin (H&E) for microscopic observation of tissue pathological changes. A control group was inoculated intraperitoneally with PBS.

### Evaluation of the immunogenic effect of Δ*pal* in mice

Thirty-two female KM mice, aged 6 to 8 weeks, were divided into an immunized group and a control group, and inoculated orally with Δ*pal*, sixteen mice per group. The procedure was as follows: mice were fasted from food and water for 7 h, orally administered 0.3 M NaHCO_3_ (200 μL/mice) to neutralize stomach acid 2 h prior to inoculation with Δ*pal*, at a dosage of 1.0 × 10^8^ CFU/mice. A booster immunization was administered 14 days after the first immunization at the same dosage of 1.0 × 10^8^ CFU/mice. The control group was inoculated orally with PBS. The immunogenic effect of Δ*pal* was evaluated based on the results of various indicators measured.

#### Detection of IgG and IgA

At days 12 and 26 post-immunization, 5 mice from each group were selected. After anesthetizing the mice, blood was collected from the eyeball and serum was separated. Sluble antigens from strain C50336 were prepared. Using 250 ng/well of these soluble antigens as coating antigens, plates were incubated at 37°C for 1 h and then overnight at 4°C. The next day, plates were washed five times with PBS containing 0.5% Tween (PBST) and blocked with 5% skim milk in PBS at 37°C for 2 h. After another five PBST washes, mouse sera collected at the 12th and 26th day post immunization, and from the control group (diluted 1:50), were added as primary antibodies and incubated at 37°C for 1 h. Following five PBST washes, HRP-conjugated goat anti-mouse IgG (diluted 1:4000) was added as the secondary antibody and incubated for another hour at 37°C. After five more PBST washes, 100 μL of TMB substrate solution (Solarbio, China) was added to each well and incubated for 10 min in the dark at 37°C. The reaction was stopped by adding 50 μL of 2 M H_2_SO_4_ to each well, and the OD_450_ was measured within 15 min.

At days 12 and 26 post-immunization, 3 mice from each group were selected. Feces were collected and resuspended in 500 μL PBS (containing 100 μg/mL soybean trypsin inhibitor (Sigma), 10 mg/mL BSA (Sigma), and 30 mM disodium EDTA, pH 7.6). After centrifugation at 4°C, the supernatant was used as the primary antibody. HRP-labeled goat anti-mouse IgA (diluted 1:2000) was used as the secondary antibody. The remaining steps followed the same procedure as the IgG ELISA assay.

#### Splenic lymphocyte proliferation assay

Mice from the control and immunized groups on the 12th and 26th day post immunization were anesthetized for blood collection via orbital bleeding and subsequently euthanized to harvest the spleen, 3 mice per group. Spleen cells were isolated in RPMI-164 medium containing 10% FBS, centrifuged, and red blood cells lysed. After neutralizing the lysis and centrifugation, cells were resuspended in RPMI-164 medium containing 10% FBS for cell counting. Cells were seeded in a 96-well plate at a density of 5 × 10^5^ cells/well, with groups for stimulation (strain C50336 soluble antigen at 100 ng/μL, 11 μL/well), non-stimulation, and medium alone, incubated for 72 h. MTT solution (5 mg/mL, Beyotime, China) was added, incubated for 4 h, followed by the addition of Formazan solvent, and incubated for another 4 h to measure OD_570_. The stimulation index (SI) was calculated using the formula (Rana and Kulshreshtha, 2006): SI = (OD of protein-stimulated group – OD of medium group)/(OD of non-stimulated group – OD of medium group).

#### Cytokine expression level detection in spleen cells

At days 14 and 28 post-immunization, spleens were collected from 3 mice in each group. Spleens of mice on the 14th and 28th day post immunization were harvested post-euthanasia and ground under cold conditions. Total RNA was extracted using the Trizol method, DNA removed, and reverse transcribed into cDNA. qPCR was performed to detect the expression levels of cytokines IL-1β, IL-2, IL-4, IL-6, IL-10, TNF-α, and IFN-γ, with primer sequences listed in Table 4. GAPDH served as the internal reference gene, and relative cytokine expression levels were calculated using the 2^−ΔΔCt^ method.

**Table 4:** Primers used for the qPCR detection of cytokines.

Primers	Primers sequences (5′ → 3′)
IL-1β-F	GACTGTTTCTAATGCCTTCCC
IL-1β-R	ATGGTTTCTTGTGACCCTGA
IL-2-F	TGAGCAGGATGGAGAATTACAGG
IL-2-R	GTCCAAGTTCATCTTCTAGGCAC
IL-4-F	GGTCTCAACCCCCAGCTAGT
IL-4-R	GCCGATGATCTCTCTCAAGTGAT
IL-6-F	TAGTCCTTCCTACCCCAATTTCC
IL-6-R	TTGGTCCTTAGCCACTCCTTC
IL-10-F	CTTACTGACTGGCATGAGGATCA
IL-10-R	GCAGCTCTAGGAGCATGTGG
IFN-γ-F	ATGAACGCTACACACTGCATC
IFN-γ-R	CCATCCTTTTGCCAGTTCCTC
TNF-α-F	CCCTCACACTCAGATCATCTTCT
TNF-α-R	GCTACGACGTGGGCTACAG
GAPDH-F	AGGTCGGTGTGAACGGATTTG
GAPDH-R	TGTAGACCATGTAGTTGAGGTCA

### Determination of the immunoprotective efficacy of Δ*pal* in mice

Thirty female KM mice, aged 6 to 8 weeks, were divided into an immunization group, a challenge group, and a control group. The immunization group was orally administered Δ*pal* at a dose of 1.0 × 10^8^ CFU/mice, followed by a booster immunization 14 days later with the same dosage. The challenge and control groups were both administered PBS orally. Twenty-eight days after the initial immunization, the immunization and challenge groups were intraperitoneally injected with a lethal dose of strain C50336, at 5 × 10^6^ CFU/mice, whereas the control group received PBS. The mortalities were recorded every day for two weeks post-challenge, and the relative percentage of survival (RPS) was calculated using the following formula (Song et al., 2018). RPS = (1-mortality in Δ*pal* immunization group/mortality in challenge group) × 100%.

### Statistical analysis

Statistical analyses were performed using SPSS version 26.0 and GraphPad Prism version 9.5.0, with the application of one-way Analysis of Variance (ANOVA) followed by t-tests. Data are expressed as mean ± standard error. Significant differences are denoted with an asterisk (*), where *p < 0.05, **p < 0.01, and ***p < 0.001 are considered to represent statistically significant differences in mean values.

## Results

### The schematic diagram of the main research conclusions

**Figure 2:**
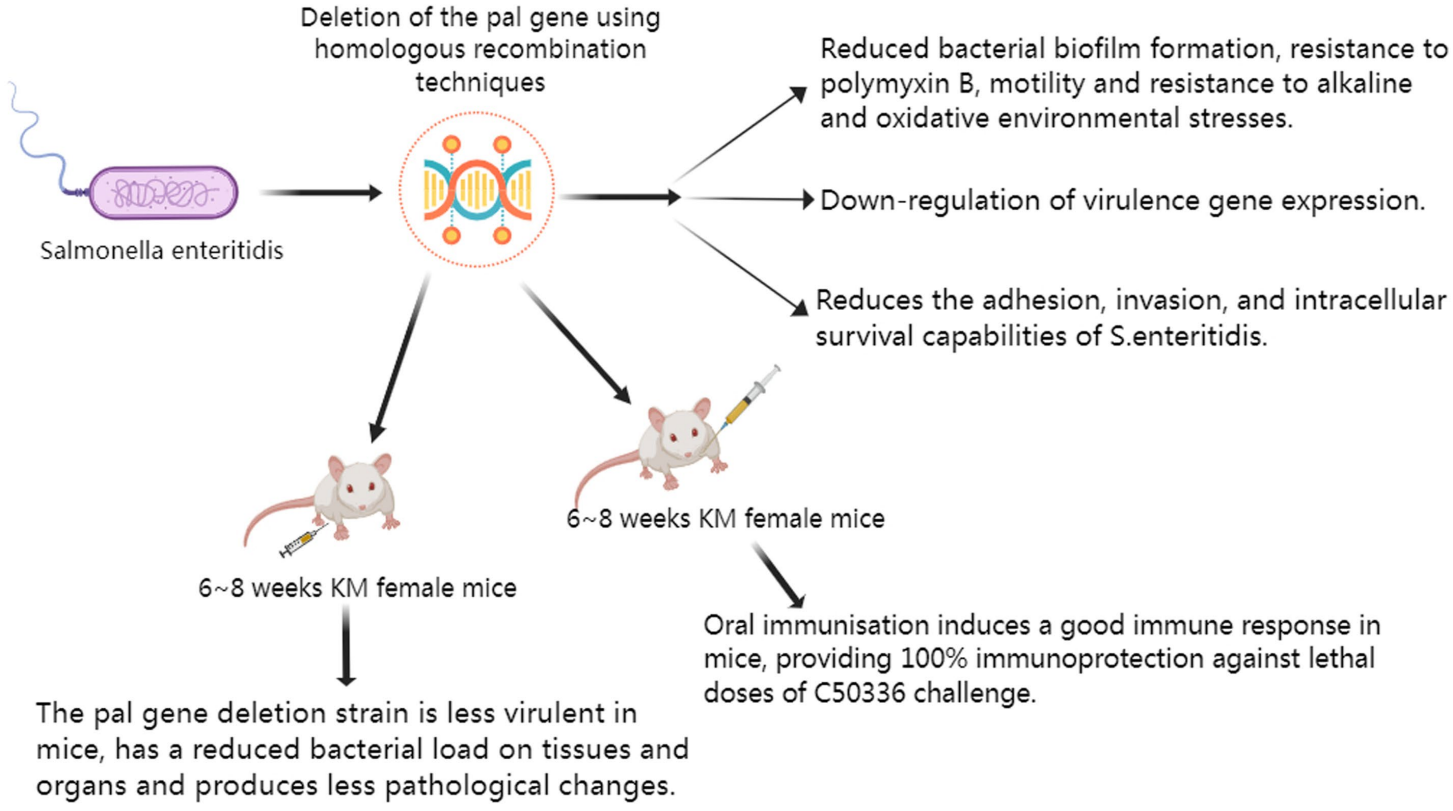
The main research conclusions.

This study evaluated the virulence of Δ*pal* through *in vitro* and *in vivo* experiments, and conducted immune protection research using mice. The main research findings are presented in the schematic diagram (Figure 2).

### Construction of *S. enteritidis* pal gene deletion and complemented strains

**Figure 3:**
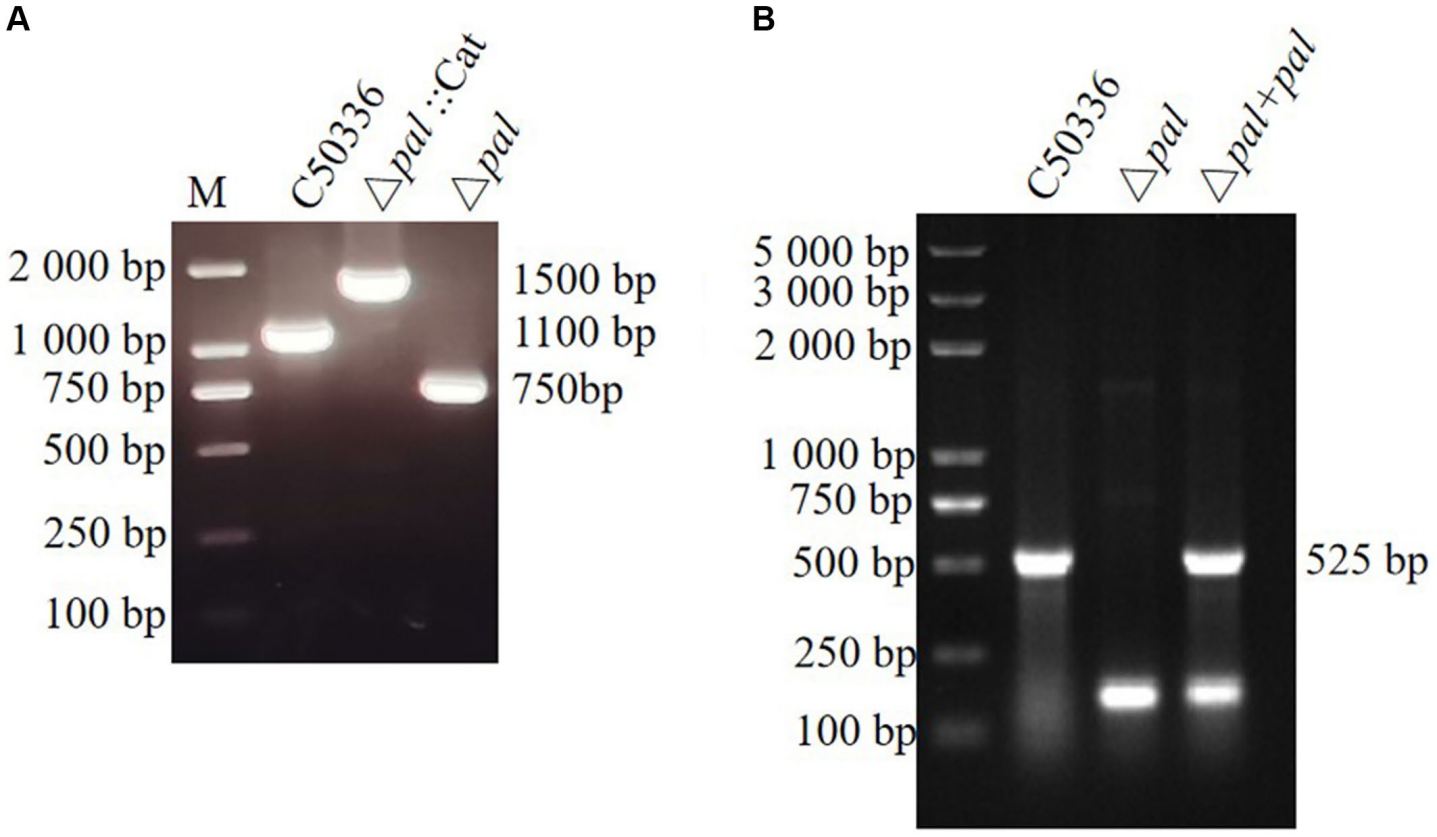
**(A)** PCR verification of the *pal* gene deletion strain and the complemented strain. C50336 represents the wild-type strain; Δ*pal::cat* represents a first recombination strain; Δ*pal* represents the *pal* deletion strain of C50336; Δ*pal* + *pal* represents the complemented strain. The PCR product of C50336 has a length of 1,100 bp. The product of Δ*pal::cat* has a length of 1,500 bp. The product of Δ*pal* has a length of 750 bp. **(B)** The product of Δ*pal* + *pal* has a length of 525 bp.

This study established a *S. enteritidis pal* gene deletion strain (Δ*pal*) and created a complemented strain (Δ*pal* + *pal*) by electroporating the recombinant plasmid pMD-19 T-*pal* into the competent cells of the *S. enteritidis pal* gene deletion strain. Primers P5 and P6 were used to amplify strain C50336, Δ*pal*::Cat, and Δ*pal*, yielding electrophoresis bands of 1,100 bp, 1,500 bp, and 750 bp, respectively. The amplification products were further sent for sequencing at a biotechnology company, and the sequencing results confirmed that Δ*pal* lacked the *pal* gene. Amplification with primers P3 and P4 of strain C50336, Δ*pal*, and Δ*pal* + *pal* produced electrophoresis bands of 525 bp (Figures 3A,B), consistent with expectations that Δ*pal* lacks the *pal* gene while Δ*pal* + *pal* contains the *pal* gene. qPCR analysis revealed that the *pal* gene was not expressed in Δ*pal*, whereas it was normally expressed in strain C50336 and Δ*pal* + *pal*. These results indicate the successful construction of Δ*pal* and Δ*pal* + *pal* in this study.

### Deletion of the pal gene reduces the biofilm formation ability of *S. enteritidis*

**Figure 4:**
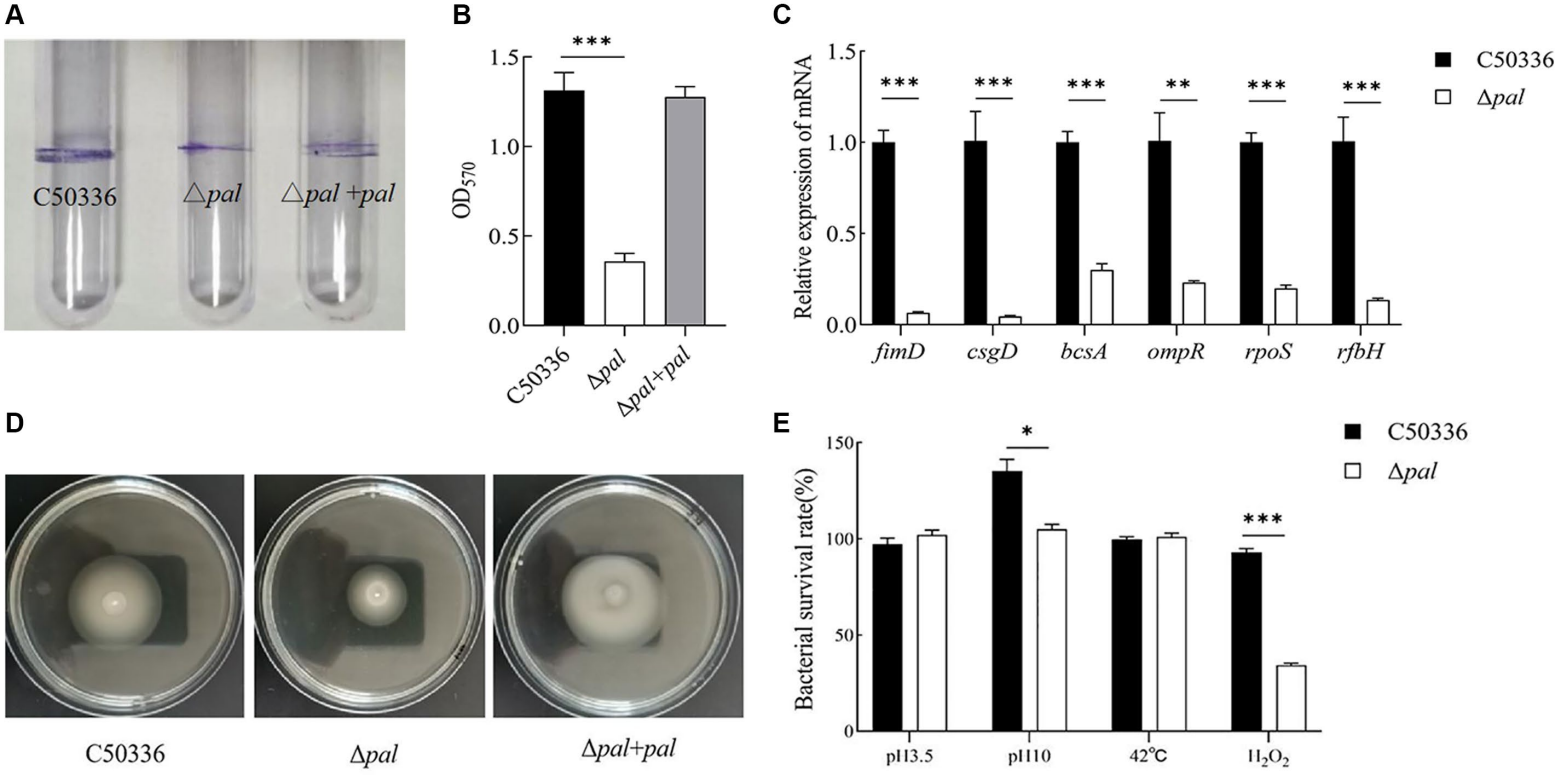
**(A)** The biofilm was assessed using tube method. **(B)** The biofilm formation ability evaluated using a 96-well plate method. **(C)** The expression analysis of biofilm-related genes was conducted by qPCR. **(D)** The motility was evaluated on 0.3% agar plates. The culture of C50336, Δ*pal*, and Δ*pal* + *pal* were inoculated vertically into LB medium containing 0.3% agar and incubated at 37°C for 6 h. The diameter of the bacterial pellicle was observed and measured. **(E)** The survival rate of Δ*pal* under various environmental stresses. C50336 and Δ*pal* were cultured in saline at pH 3.5 and pH 10 for 1 h, in saline containing H_2_O_2_ for 10 min, and at 42°C for 1 h, respectively. Bacterial counts were performed, and bacterial survival rates were calculated (*n* = 3, *p < 0.05, **p < 0.01, ***p < 0.001).

The biofilm formation ability of Δ*pal* was assessed using the tube method, and the results (Figure 4A) showed that compared to C50336 and Δ*pal* formed a significantly thinner biofilm. Results from the 96-well plate method (Figure 4B) showed that the OD_570_ of strain C50336 was 1.313 (±0.2), while that of Δ*pal* was 0.358 (±0.2). Compared to strain C50336, the biofilm formation ability of Δ*pal* decreased by 73%. The expression levels of biofilm-related factors (Figure 4C) showed that the expression of *fimD*, *csgD*, *bcsA*, *ompR*, and *ropS* genes in Δ*pal* were downregulated compared to strain C50336, indicating that the *pal* gene reduces the biofilm formation ability of *S. enteritidis*.

### Deletion of the pal gene reduces the drug resistance of *S. enteritidis*

Polymyxin B serves as a last line of defense in the treatment of antibiotic-resistant Gram-negative bacteria. It functions by enhancing the permeability of the cell membrane, leading to the leakage of cellular contents and ultimately resulting in bacterial death. Consequently, polymyxin B can be employed to assess bacterial resistance. In this study, polymyxin B was used to analyze the resistance of C50336 and Δ*pal*. The results (Table 5) show that the MIC of polymyxin B against C50336 was 7.813 μg/mL, while it was 1.95 μg/mL against Δ*pal*. The deletion of the *pal* gene resulted in a 75% reduction in the MIC of polymyxin B against *S. enteritidis*, indicating that the deletion of the *pal* gene reduces the drug resistance of *S. enteritidis*.

**Table 5:** MIC experiments of C50336 and Δ*pal* against polymyxin B.

Strains/Dilution	2^0^	2^1^	2^2^	2^3^	2^4^	2^5^	2^6^	2^7^	2^8^	2^9^	2^10^	2^11^	Control group without polymyxin B
C50336	−	−	−	−	−	−	−	−	+	+	+	+	+
Δ*pal*	−	−	−	−	−	−	−	−	−	−	+	+	+

### Deletion of the pal gene reduces the motility of *Salmonella enteritidis*

The motility was assessed by measuring the diameter of bacterial movement on LB agar containing 0.3% agar, and the results (Figure 4D) showed that the motility diameters of strain C50336, Δ*pal*, and Δ*pal* + *pal* within 6 h were 38 mm, 28 mm, and 39 mm, respectively. This indicates that deletion of the *pal* gene can reduce the motility of *S. enteritidis*.

### The pal gene affects the resistance of *S. enteritidis* to environmental stress

To study whether the *pal* gene influences the resistance of *S. enteritidis* to various environmental stresses, we compared the survival of C50336 and Δ*pal* in strong acid stress solution, strong alkaline stress solution, at 42°C, and in oxidative stress solution. The results (Figure 4E) showed that compared to strain C50336, the survival rate of Δ*pal* significantly decreased (*p* < 0.05) in strong alkaline stress solution and significantly decreased (*p* < 0.001) in oxidative stress solution, while there was no significant change in survival rate in strong acid stress solution and at 42°C. These results indicate that deletion of the *pal* gene can reduce the antioxidant capacity and survival ability of *S. enteritidis* in oxidative stress solution.

### Deletion of the pal gene reduces the adhesion, invasion, and intracellular survival capabilities of *S. enteritidis*

**Figure 5:**
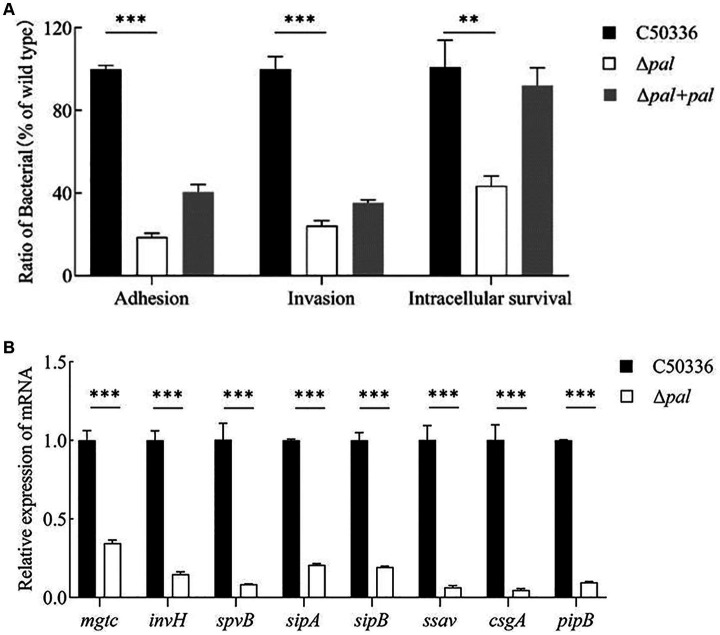
**(A)** Adhesion and invasion and intracellular survival experiments. Caco-2 cells were used to perform the adhesion and invasion experiments, and RAW 264.7 cells were used to perform the intracellular survival experiments. The results are presented as a ratio to the C50336. The results of C50336 were considered as 100%. **(B)** The expression analysis of virulence genes in C50336 and Δ*pal* were conducted by qPCR, with 16S rRNA as the reference gene (*n* = 3, *p < 0.05, **p < 0.01, ***p < 0.001).

Adhesion experiments conducted with the human colorectal carcinoma epithelial cell line Caco-2 showed that (Figure 5A), compared to strain C50336, the adhesion rate of Δ*pal* to Caco-2 cells significantly decreased (*p* < 0.001), about 18% of that of strain C50336, and the adhesion capability of Δ*pal* + *pal* was restored to about 40%. These results indicate that deletion of the *pal* gene reduces the ability of *S. enteritidis* to adhere to intestinal epithelial cells.

Invasion experiments using the Caco-2 cell line demonstrated that (Figure 5A), compared to the wild-type strain C50336, the invasion rate of Δ*pal* significantly decreased (*p* < 0.001), about 24% of that of C50336, and the invasion capability of Δ*pal* + *pal* was restored to about 35%. This indicates that deletion of the *pal* gene reduces the ability of *S. enteritidis* to invade intestinal epithelial cells.

Intracellular survival experiments conducted with the murine macrophage cell line RAW264.7 showed that (Figure 5A), compared to C50336, the intracellular survival capability of Δ*pal* significantly decreased (*p* < 0.01), about 40% of that of C50336, and the intracellular survival capability of Δ*pal* + *pal* was restored to about 80%. These results suggest that deletion of the *pal* gene reduces the survival ability of *S. enteritidis* in macrophages.

### Reduced expression levels of virulence genes in pal gene deletion strain

To elucidate the mechanism by which the *pal* gene affects the virulence of *S. enteritidis*, qPCR was utilized to assess the expression levels of various virulence genes in C50336 and Δ*pal*. The results (Figure 5B) show that compared to strain C50336, the expression levels of virulence genes such as *mgtc*, *invH*, *spvB*, *sipA*, *sipB*, *ssav csgA*, and *pipB* in Δ*pal* were significantly downregulated (*p* < 0.001). These findings indicate that deletion of the *pal* gene reduces the expression levels of virulence genes in *S. enteritidis*.

### Deletion of the pal gene attenuates the virulence of *S. enteritidis*

Female KM mice, aged 6 to 8 weeks, were inoculated intraperitoneally with C50336 and Δ*pal*. The health condition and mortality of the mice were observed daily for a continuous period of 14 days. The results (Table 6) show that the LD_50_ of strain C50336 was 6.3 × 10^5^ CFU/mouse, whereas the LD_50_ of Δ*pal* was 5.6 × 10^9^ CFU/mouse, making the LD_50_ of Δ*pal* approximately 10^4^ times greater than that of C50336 (5.6 × 10^9^/6.3 × 10^5^ ≈ 10^4^). These results indicate that deletion of the *pal* gene attenuates the virulence of *S. enteritidis*.

**Table 6:** LD_50_ of C50336 and Δ*pal.*

Strains	Infection dose (CFU/mouse)	Number of dead mice/Number of total mice	LD_50_ (CFU/mouse)
C50336	2.0 × 10^7^	5/5	6.3 × 10^5^
	2.0 × 10^6^	3/5	
	2.0 × 10^5^	2/5	
	2.0 × 10^4^	0/5	
	2.0 × 10^3^	0/5	
Δ*pal*	1.6 × 10^10^	4/5	5.6 × 10^9^
	1.6 × 10^9^	1/5	
	1.6 × 10^8^	0/5	
	1.6 × 10^7^	0/5	
	1.6 × 10^6^	0/5	

### Deletion of the pal gene reduces the colonization ability of *S. enteritidis* in mice

**Figure 6:**
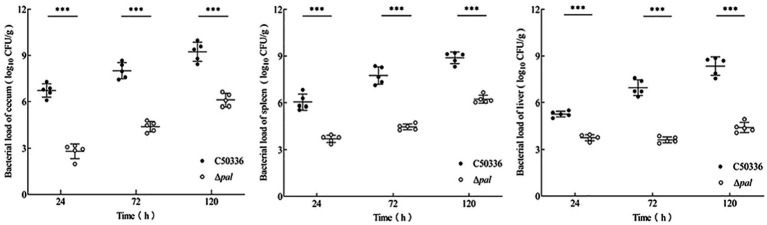
The bacterial load of C50336 and Δ*pal* in mouse tissues or organs. At 24 h, 72 h, and 120 h post-intraperitoneal injection of C50336 and Δ*pal* into mice, the cecum, liver, and spleen were harvested and homogenized. The drop plate counting methods were used to calculate the bacterial load in tissues or organs (*p < 0.05, **p < 0.01, ***p < 0.001).

Mice were infected with equivalent doses of strain C50336 and Δ*pal* for the same duration, and an analysis of bacterial load in tissue organs was conducted. The results (Figure 6) show that at 24, 72, and 120 h post infection, the bacterial loads of Δ*pal* in the jejunum, spleen, and liver were significantly lower than those of C50336. After infection with Δ*pal*, up to 120 h, the bacterial loads in the cecum and spleen reached about 10^6^ CFU/g, and the bacterial load in the liver never reached 10^6^ CFU/g. In contrast, within 24 h of infection with strain C50336, the bacterial loads in the cecum and spleen had already reached 10^6^ CFU/g; at 72 and 120 h, the bacterial loads in the cecum, spleen, and liver all exceeded 10^6^ CFU/g. These results indicate that *S. enteritidis* with the *pal* gene deleted can still colonize in the organs of mice, but its colonization ability is significantly reduced compared to C50336.

### Mice immunized with Δ*pal* did not exhibit significant clinical symptoms or pathological changes

**Figure 7:**
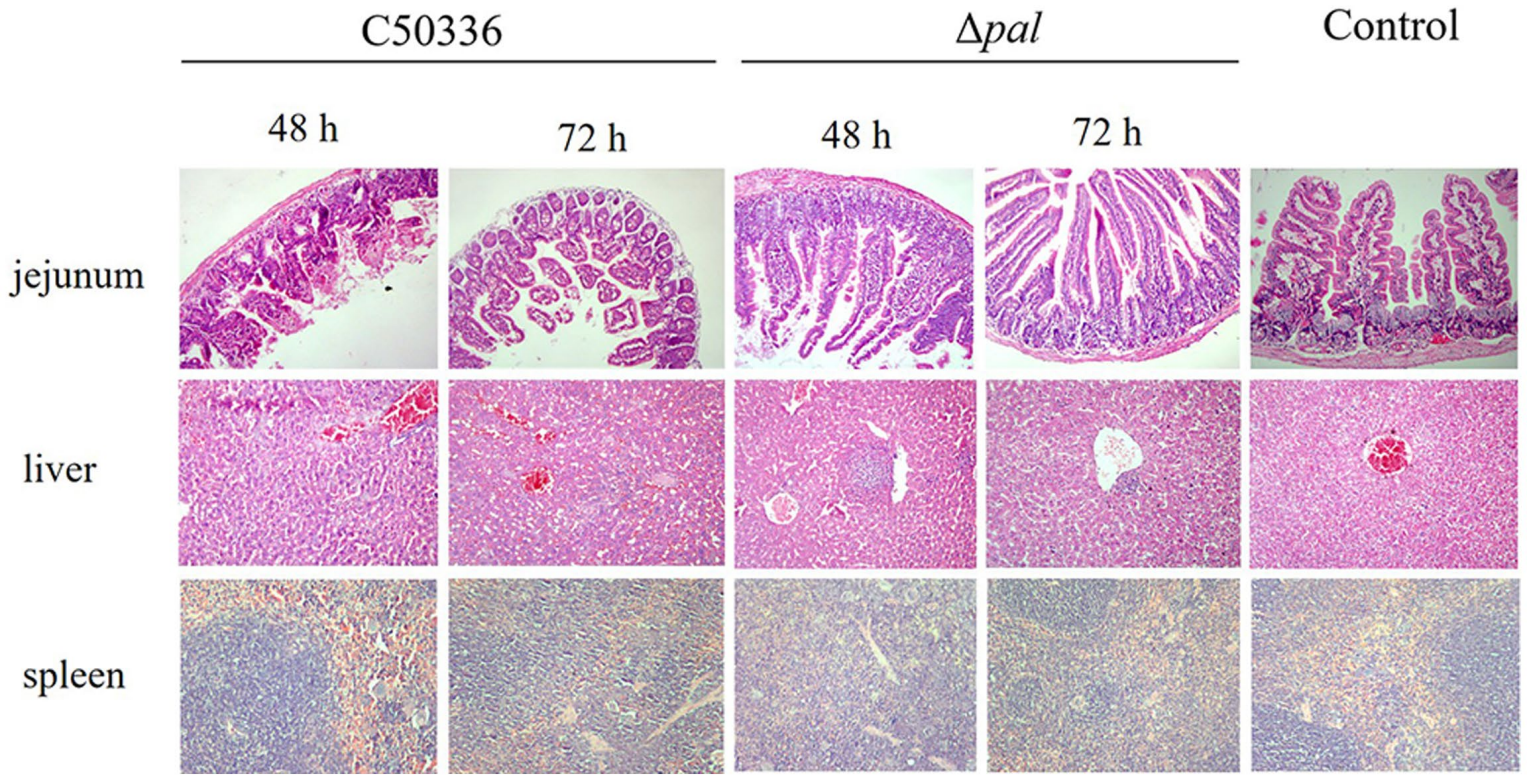
Histopathological images of mouse tissues infected with C50336 and Δ*pal*. At 48 and 72 h post infection with C50336 and Δ*pal*, sections of the mouse jejunum, liver, and spleen were fixed in 4% paraformaldehyde, embedded in paraffin, sectioned, and stained with hematoxylin and eosin (H&E). Microscopic observations were conducted (200×).

The results (Figure 7) indicate that, at 48 h post-infection with C50336, there was evident destruction of the jejunum villi and extensive infiltration of red blood cells. At 72 h post-infection, the jejunum villi were shed and became sparse, with damaged epithelium; however, Δ*pal* did not cause significant damage to the mouse jejunum. At 48 h post-infection with C50336, the liver tissue structure was unclear, the central vein was dilated and congested, and hepatocytes showed vacuolization. By 72 h post-infection, sinusoidal dilatation filled with red blood cells and severe hepatocyte vacuolization were observed; whereas, at 48 and 72 h post infection with Δ*pal*, the liver tissue structure was clear, with focal necrosis in a few hepatocytes and lymphocyte aggregation visible. At 48 h post-infection with C50336, the spleen showed dilated and congested splenic sinuses containing large amounts of red blood cells. By 72 h, there was no clear demarcation between red and white pulp, and the splenic cord structure was unclear. In contrast, mice infected with Δ*pal* did not exhibit significant pathological changes in the spleen.

### StrainΔ*pal* can induce the production of specific IgG and IgA antibodies in mice

**Figure 8:**
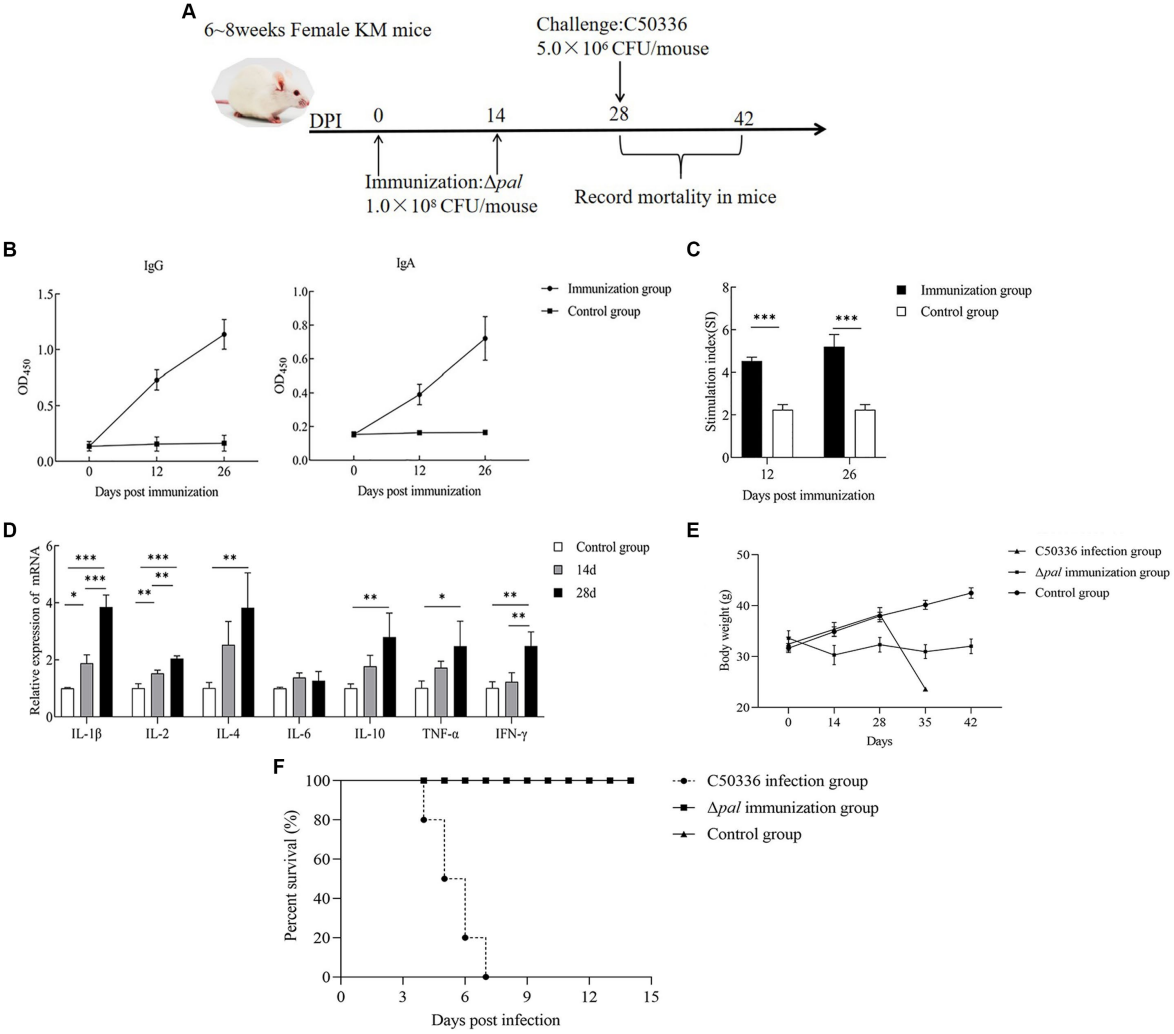
Immunological parameters determination. **(A)** Immunization process diagram of female KM mice aged 6 to 8 weeks. **(B)** KM mice orally vaccinated with Δ*pal*, using ELISA to determine the levels of IgG and IgA in mice at days 12 and 26 post-immunization. **(C)** Measurement of lymphocyte proliferation and transformation levels in the spleen of mice at days 12 and 26 post-immunization using the MTT assay, calculating the stimulation index (SI). **(D)** Measurement of cytokine expression levels in the spleen tissue of mice at days 14 and 28 post-immunization using qPCR. **(E)** Changes in body weight of mice after immunization with Δ*pal*. **(F)** Survival curves of mice in the immunized group, challenged group, and control group after intraperitoneal infection with C50336 within 14 days. *p < 0.05, **p < 0.01, ***p < 0.001.

By immunizing 6-8-week-old KM mice with Δ*pal* on days 0 and 14 (Figure 8A), mouse serum and feces were collected on the 12th and 26th day post immunization for IgG and IgA antibody detection. The results (Figure 8B) demonstrate that, at 12 days post immunization, the levels of IgG antibodies in mice were significantly higher than those in the control group, with antibody levels further increasing by the 26th day post immunization. This indicates that Δ*pal* can induce a humoral immune response in mice. The detection of IgA antibodies (Figure 8B) revealed that, on the 12th day post immunization, fecal IgA levels were significantly higher than those in the control group, with IgA levels further elevated on the 26th day post immunization. This suggests that Δ*pal* can induce a mucosal immune response in mice.

### StrainΔ*pal* enhances cellular immune levels in mice

Mice splenocyte on the 12th and 26th day post immunization was harvested for lymphocyte proliferation assays. The results (Figure 8C) indicated that the stimulation indices (SI) for the immunized group were 4.532 ± 0.184 (12 days) and 5.212 ± 0.571 (26 days), significantly higher than those of the control group (2.249 ± 0.242). Furthermore, the SI on the 26th day post immunization was higher than that at 12 days post-immunization. These findings suggest that Δ*pal* significantly enhances the proliferative transformation capacity of splenic lymphocytes, elevating the specific cellular immune levels in mice. Moreover, the level of cellular immunity was further increased with additional rounds of immunization.

### StrainΔ*pal* upregulates cytokine expression levels in mouse spleens

The expression of cytokines in mouse spleens on the 14th and 28th day post immunization was measured using the qPCR method. The results (Figure 8D) showed that, on the 14th and 28th day post immunization, the expression levels of IL-1β, IL-2, and IFN-γ were significantly higher than those in the control group. Furthermore, at 28 days post-immunization, the expression levels of these cytokines, including pro-inflammatory cytokines, were significantly higher than at 14 days post-immunization. On the 14th and 28th day post immunization, the expression levels of IL-4, IL-10, and TNF-α were significantly higher than those in the control group, but there was no significant difference in the cytokine levels between 14 and 28 days. The expression levels of IL-6 did not show significant changes at different time points post-immunization. These results indicate that Δ*pal* can upregulate the expression levels of cytokines including IL-1β, TNF-α, IL-2, IL-4, and IL-10, suggesting that Δ*pal* stimulates a strong immune response in mice.

### StrainΔ*pal* provides effective immune protection in mice

At 28 days post-immunization, mice in both the immunized and control groups were administered a lethal dose of C50336 via intraperitoneal injection, with a dosage of 5.0 × 10^6^ CFU/mouse. The incidence of disease and mortality were recorded daily for a continuous observation period of 14 days. Mice in the challenge group exhibited typical symptoms of *S. enteritidis* infection, such as trembling, arched backs, crusted eyes, and disheveled fur, starting on day 3, with deaths occurring from day 4 and reaching a mortality rate of 100% by day 7. In contrast, mice in the immunized group showed only mild adverse reactions within the first 3 days post-challenge, including reduced food intake and slightly disheveled fur, but subsequently recovered to normal, with no deaths occurring within 14 days. After the first immunization with Δ*pal*, the mice’s body weight initially decreased and then increased. When C50336 was administered at 28 days post-immunization, the body weight of the immunized mice slightly decreased before increasing again (Figure 8E). The results (Figure 8F) demonstrated a relative survival rate of 100% in the immunized group, indicating that Δ*pal* provides effective immune protection against strain C50336 challenge in mice.

## Discussion

*S. enteritidis* is a zoonotic pathogen that severely endangers public health safety. Vaccine immunization is an effective measure to prevent and control *Salmonella* infection. Attenuated live vaccines can promote a strong cellular immune response, which is more conducive to the clearance of intracellular parasitic pathogens. Pal is an important lipoprotein in the biofilm matrix; deletion of the *pal* gene can affect the formation of biofilms by *Haemophilus ducreyi*, but the impact of the *pal* gene on the biofilm formation ability of *S. enteritis* is not yet clear. Therefore, this study constructed an *S. enteritidis pal* gene deletion strain to test the biofilm formation ability of Δ*pal*. It was found that deletion of the *pal* gene significantly reduced the biofilm formation ability of *S. enteritidis*, consistent with results from *Haemophilus ducreyi* research. Analysis of the expression of biofilm-related genes found that the expression levels of *fimD*, *csgD*, *bcsA*, *ompR*, *ropS*, and *rfbH* genes in the *pal* gene deletion strain were downregulated, further explaining the mechanism by which the *pal* gene affects biofilm formation. Deletion of the *pal* gene significantly reduces the biofilm formation ability of *S. enteritidis*. Biofilms are often associated with bacterial drug resistance and motility. As an outer membrane lipoprotein, the absence of Pal might affect changes in the outer membrane components of *S. enteritidis*, potentially affecting changes in bacterial drug resistance and motility. Since polymyxin B is a broad-spectrum antimicrobial peptide commonly used to treat Gram-negative bacterial infections, this study analyzed the drug resistance of the *S. enteritidis pal* gene deletion strain with polymyxin B and found that Δ*pal* showed reduced drug resistance, consistent with conjectural results. *Salmonella* has motility mediated by flagella, which is an important characteristic of its high virulence. Therefore, the motility of the *S. enteritidis pal* gene deletion strain was analyzed, showing reduced motility in Δ*pal* compared to strain C50336, consistent with findings by Zhou X and others that *Helicobacter pylori pal* gene deletion strains move more slowly (Solanki et al., 2023). Combined, these results suggest Pal might affect the virulence of *S. enteritidis*.

Since *Salmonella* is transmitted via the fecal-oral route, bacteria pass through the stomach and then colonize the intestines, resisting digestion by gastric and intestinal fluids, entering the submucosal layer of the intestines, being phagocytosed by phagocytic cells, and surviving within phagocytic cells before ultimately spreading throughout the body to complete colonization and infection. Hence, *Salmonella* has evolved over a long period to possess strong resistance to environmental stimuli such as acid, alkali, and oxidation. This study found that Δ*pal* has significantly reduced antioxidant capacity and resistance to alkaline environments, indicating the *pal* gene is involved in *S. enteritidis ‘s* defense against stress, thereby promoting bacterial survival within the host. Phagosomes formed by phagocytic cells during the phagocytosis of bacteria provide a strong oxidative environment and other adverse stresses to eliminate pathogens within cells. Therefore, it is reasonable to believe that the survival ability of Δ*pal* within phagocytic cells will be significantly reduced, thereby affecting bacterial virulence. This study used the murine macrophage line RAW264.7 for phagocytosis experiments, finding that the survival rate of Δ*pal* within macrophages is significantly lower than the wild type, thus proving the aforementioned conjecture.

The *pal* gene encodes a peptidoglycan-associated lipoprotein; its deletion might affect the formation of biofilms. This study confirmed that the biofilm formation ability of *S. enteritidis pal* gene deletion strain is significantly reduced. The formation of biofilms is associated with bacterial invasion and adhesion, and a diminished capacity for biofilm formation could result in alterations in bacterial infection potential. Therefore, this study used Δ*pal* for invasion and adhesion experiments on Caco-2 cells, finding that deletion of the *pal* gene significantly reduces the adhesion and invasion abilities of *S. enteritidis*. Solanki V and others found that deletion of the *pal* gene could reduce the adhesion and invasion abilities of *Acinetobacter baumannii* to host lung epithelial cells, similar to the results of this study (Zhou et al., 2024).

The *pal* gene affects *S. enteritidis* ‘s adhesion to cells, invasion, and phagocytic killing, influencing the bacterium’s virulence at the cellular level. To evaluate the virulence of Δ*pal* at the animal infection level, this study measured LD_50_, bacterial loads in mouse organs, and histopathological changes, finding that the LD_50_ of Δ*pal* is about 10^4^ times greater than the wild type, indicating significantly reduced virulence of Δ*pal*; and after infection for 24 h, 72 h, and 120 h, the bacterial loads in the spleen, liver, and cecum of the *pal* gene deletion strain were significantly lower than those of the wild type. Chen Y and others found that deletion of the *pal* gene in *Brucella* reduced bacterial colonization ability in the host spleen, consistent with this study (Chen et al., 2022). Therefore, it can be confirmed that deletion of the *pal* gene weakens the colonization ability of *S. enteritidis* in host tissues, making it easier for the immune system to eliminate.

*Salmonella* encodes many virulence factors that can affect its survival ability within macrophages and regulate its adhesion and invasion abilities to intestinal epithelial cells. The virulence factor MgtC protein not only promotes bacterial proliferation within macrophages but is also an important virulence factor of *Salmonella* (Lee and Lee, 2015); the virulence factor SpvB has ADP-ribosyltransferase activity, which can inhibit G protein assembly, ultimately leading to cytoskeletal depolymerization; the virulence factor InvH is a transmembrane protein, and the virulence factor Ssav is the largest component of the *Salmonella* type III secretion system needle complex, closely related to bacterial entry into the host. Deleting virulence genes *invH* and *ssaV* can reduce *Salmonella*’s invasion ability to epithelial cells and intestines, delaying the intestinal inflammatory response to *Salmonella* infection and reducing the bacterial load in mucosal tissues (Pati et al., 2013); virulence factors SipA and SipB are invasion proteins, and deleting the *sipA* gene can significantly reduce *Salmonella*’s adhesion and invasion abilities to epithelial cells; the virulence factor PipB is related to bacterial intracellular survival, and deleting the *pipB* gene reduces *Salmonella*’s survival ability within macrophages; the virulence factor CsgA is related to bacterial adhesion and invasion abilities. Since Δ*pal* shows reduced adhesion, invasion, and intracellular survival abilities to epithelial cells, to analyze its molecular mechanism, this study used qPCR to measure the expression levels of virulence genes *mgtc*, *spvB*, *invH*, *pipB*, *ssav*, and *csgA*, finding that, compared to the wild-type strain, the expression levels of these virulence genes in Δ*pal* are significantly downregulated, which to some extent explains the mechanism of reduced virulence in Δ*pal*.

To further evaluate the safety of Δ*pal* as a candidate attenuated live vaccine, histopathological evaluations of the spleen, liver, and jejunum of mice infected with the same dose of C50036 and Δ*pal* at the same time were conducted. Histopathological slice results showed that with increasing infection time, the spleen, liver, and jejunum of mice infected with the wild type underwent severe pathological changes, while the pathological changes in the tissues and organs of mice infected with Δ*pal* were minor, indicating Δ*pal* causes less pathological damage to mice and has good safety.

Since *S. enteritidis* is transmitted via the fecal-oral route, mucosal immunity in the intestines and other areas is extremely important for defending against pathogen infection. SIgA is a key immune molecule in mucosal immunity. Additionally, IgG in serum is an important indicator of humoral immunity in the body and plays an important role in immune defense against *S. enteritidis*. Therefore, to further evaluate the immune protection effect of Δ*pal*, this study conducted IgA and IgG tests on mice immunized with Δ*pal*, finding that with the extension of immunization time and increase in the number of immunizations, Δ*pal* can induce mice to produce high doses of SIgA and IgG. Additionally, lymphocyte proliferation ability is an important component of body immunity. Lymphocyte proliferation experiments found that the spleen cell stimulation index of the immunized group significantly increased, promoting lymphocyte proliferation and transformation. Through antibody testing and lymphocyte proliferation and transformation experiments, it was found that Δ*pal* can induce mice to produce specific humoral and cellular immunity.

Cytokines play a crucial role in evaluating the efficacy of vaccines. They are signaling molecules in the immune system that regulate and modulate the occurrence and intensity of immune responses. IL-1β, IL-6, and TNF-α are important pro-inflammatory factors in the body (Song et al., 2022), while IL-10 is an important anti-inflammatory and immune-regulating cytokine (Albuquerque Pereira et al., 2023), playing an important role in alleviating immune damage caused by infections. IL-2and IL-4 are involved in regulating immune response reactions, playing important roles in host cellular and humoral immunity (Ullrich et al., 2021; Kung et al., 2022), respectively; IFN-γ plays a key role in defending against intracellular bacterial infections (Kak et al., 2018). The study results showed that the expression levels of the above cytokines were increased to varying degrees compared to the unimmunized group, indicating that Δ*pal* as a candidate attenuated live vaccine can effectively enhance the body’s immune level.

Finally, this study conducted a lethal dose protection experiment using KM mice, finding that immunized mice can resist attacks from the lethal dose of the wild-type strain C50336, with a relative protection rate of 100%, indicating Δ*pal* provides good immune protection for mice.

In summary, this study shows that deletion of the *pal* gene reduces the virulence of *S. enteritidis*, Δ*pal* can induce the host to produce mucosal and systemic immune responses, has good safety, and provides good immune protection for mice against *S. enteritidis* infection, making it a candidate for an attenuated live vaccine for *S. enteritidis*. Nevertheless, the safety profile of Δ*pal* as a vaccine remains incompletely elucidated. For instance, it is unclear whether Δ*pal* can be completely cleared from the host following immunization of animals with Δ*pal*. Additionally, the target animal for the development of the Δ*pal* vaccine against *S. enteritidis* infection is not clearly defined. Furthermore, commercial vaccines were not utilized as controls in the vaccine evaluation trials.

## Data availability statement

The original contributions presented in the study are included in the article/supplementary material, further inquiries can be directed to the corresponding author.

## Ethics statement

The animal study was approved by the Animal Ethics Committee of Hebei Normal University of Science and Technology (2020-17). The study was conducted in accordance with the local legislation and institutional requirements.

## Author contributions

GZ: Data curation, Formal analysis, Writing – original draft. WD: Methodology, Writing – original draft. LZ: Data curation, Formal analysis, Methodology, Writing – original draft. WS: Data curation, Formal analysis, Methodology, Writing – original draft. WL: Data curation, Methodology, Software, Writing – original draft. XZ: Data curation, Software, Writing – original draft. YZ: Funding acquisition, Validation, Writing – original draft. QS: Funding acquisition, Writing – review & editing. TW: Conceptualization, Funding acquisition, Investigation, Methodology, Writing – review & editing.
